# Manual acupuncture for neuromusculoskeletal disorders: The selection of stimulation parameters and corresponding effects

**DOI:** 10.3389/fnins.2023.1096339

**Published:** 2023-01-30

**Authors:** Bing-Gan Wang, Liu-Liu Xu, Hua-Yuan Yang, Jian Xie, Gang Xu, Wen-Chao Tang

**Affiliations:** ^1^School of Acupuncture-Moxibustion and Tuina, Shanghai University of Traditional Chinese Medicine, Shanghai, China; ^2^Department of Acupuncture and Moxibustion, Yuhuan Hospital of Traditional Chinese Medicine, Taizhou, Zhejiang, China

**Keywords:** manual acupuncture, stimulation parameters, therapeutic effects, review, mechanism of action

## Abstract

As a minimally invasive method of physical stimulation, manual acupuncture (MA) is used globally as a sort of therapy for neuromusculoskeletal disorders. In addition to selecting appropriate acupoints, acupuncturists should also determine the stimulation parameters of needling, such as the manipulation (lifting-thrusting or twirling), needling amplitude, velocity, and stimulation time. At present, most studies focus on acupoint combination and mechanism of MA, the relationship between stimulation parameters and their therapeutic effects, as well as the influence on mechanism of action are relatively scattered, and lack of systematic summary and analysis. This paper reviewed the three types of stimulation parameters of MA, their common options and values, corresponding effects and potential mechanisms of action. The purpose of such efforts is to provide a useful reference for the dose-effect relationship of MA and the quantification and standardization of its clinical treatment of neuromusculoskeletal disorders to further promote the application of acupuncture in the world.

## 1. Introduction

As a minimally invasive method of physical stimulation, acupuncture is widely used globally as a sort of therapy of traditional Chinese medicine (TCM) and clinical skill; according to the statistics of the World Health Organization (WHO), it has been used in 183 countries or regions around the world (World Health Organization [WHO], 2013). Acupuncture has a wide range of indications and is also recommended by WHO because of its safety, simplicity and efficacy (World Health Organization [WHO], 2019). Among these indications, the use of manual acupuncture (MA) in neuromusculoskeletal disorders has been around for hundreds of years and is very prevalent. Some ancient Chinese medical books have records of MA for the relief of low back and leg pain and the improvement of limited mobility (Zhu et al., 2021). At the same time, many modern clinical randomized controlled trials (RCT) have also confirmed the therapeutic effects of MA on these diseases (Zhang X. et al., 2019; Chen L. et al., 2021).

During the treatment process of neuromusculoskeletal disorders, in addition to selecting appropriate acupoints according to the patient’s condition and TCM theory, acupuncturists should also determine the stimulation parameters of needling, such as the selections of acupuncture manipulation (lifting-thrusting or twirling), operation amplitude, velocity, and stimulation time (Lyu et al., 2019). Numerous studies have suggested that different stimulation methods of acupuncture can result in different needling sensations and therapeutic effects. For instance, the needling sensation caused by lifting-thrusting is usually stronger than that of twirling (Huang et al., 2012), whereas twirling may further improve the patient’s pressure pain threshold (Choi et al., 2013). Meanwhile, even with the same acupuncture manipulation, some studies found that a better analgesic effect could be achieved by greater needling velocity (Yin et al., 2011) or amplitude (Itoh et al., 2011). In addition, the persistence of such sensation and pain-relieving effects is also based on a certain time of stimulation. Comparative studies found that manipulation for a certain period of time has a better effect than simply inserting a needle into acupoints in pain relief (Loyeung and Cobbin, 2013).

Throughout the current research on MA treatment of neuromusculoskeletal disorders, most of them focus on the selection of acupoints before stimulation and the mechanism investigation after stimulation, but the relationship between stimulation parameters during treatment and their therapeutic effects, as well as the influence on mechanism of action, are relatively scattered and lack systematic summary and analysis. In contrast, electroacupuncture (EA) has received increasing attention from acupuncturists due to its four clear, quantified and easily controlled stimulation parameters (waveform, frequency, time, and current intensity) (Omura, 1987; Baba et al., 2002), and there are more RCTs comparing the effects of different stimulation parameters of EA (Liu et al., 2017; Zheng et al., 2018). For example, there are correlations between different frequencies (Humaidan et al., 2006; Yang et al., 2020; Yao S. et al., 2020), current intensities (Tamai et al., 2020), stimulation time (Heo et al., 2022) and different therapeutic effects, so dose-effect relationship studies of EA provide rich evidence and references for its clinical application. This paper reviewed the different stimulation parameters related to the effect of MA intervention, their common options and values, corresponding effects and potential mechanisms of action. The purpose of such efforts is to provide a useful reference for the dose-effect relationship of MA, and the quantification and standardization of its treatment of neuromusculoskeletal disorders to further promote the application of acupuncture in the world.

## 2. Ma effects on neuromusculoskeletal disorders and potential mechanisms

The therapeutic effects of MA on neuromusculoskeletal disorders are mainly manifested in relieving pain (Shah and Thaker, 2015), reducing local inflammatory response (Xu et al., 2018), improving limited limb mobility (Lu et al., 2010) and so on. When MA stimulates the acupoints, the tissue around the needle body receives mechanical stimulation during the needle’s movement, these tissues can further transmit mechanical stimulation to the surrounding cells and activate mechanosensitive ion channels in the cell membrane, including transient receptor potential cation channel subfamily V members (TRPV1, TRPV2, and TRPV4) (Chen et al., 2018; Huang et al., 2018; Zheng et al., 2021), Piezo proteins (Piezo1, Piezo 2) (Guo et al., 2022) and stretch-activated chloride channels (SACs) (Wang and Schwarz, 2012). Then, cellular responses such as mast cell degranulation, fibroblast activation and macrophage polarization are initialized with the ion influx (Yao et al., 2014) and release relevant active substances, including adenosine triphosphate (ATP) (Wang and Schwarz, 2012) and histamine (Huang et al., 2018; Yin et al., 2018). After these substances bind to the corresponding receptors in the nerve endings, the expression of receptors related to pain signal transmission will be downregulated and inhibit the upload of pain signals (Tang et al., 2016). At the same time, stimulation with MA also promotes the release of substance P (SP) from nerve endings, which further promotes the above process to facilitate peripheral analgesia (Gong et al., 2020; Fan et al., 2021). In the central nervous system (CNS), the uploaded acupuncture signal promotes the release of substances such as gamma-aminobutyric acid (GABA), 5-hydroxytryptamine (5-HT), epinephrine (NE), and opioid peptides (Ko et al., 2018; Lin et al., 2020; Qiao et al., 2020) and guides the descending analgesic signal to enhance the analgesic effect. The effects of MA on CNS have also been investigated by some functional neuroimaging studies, the scanning result of functional magnetic resonance imaging (fMRI) showed that the central analgesic effect of MA was based on the increase of default mode network and sensorimotor network connectivity with pain-related brain areas (Cai et al., 2018), and the equilibrium regulation of distributed pain-related central networks (Biella et al., 2001). Meanwhile, the *deqi* sensation of MA may be related to the significant deactivations of brain fMRI blood oxygen level-dependent (BOLD) signals (Asghar et al., 2010).

**FIGURE 1:**
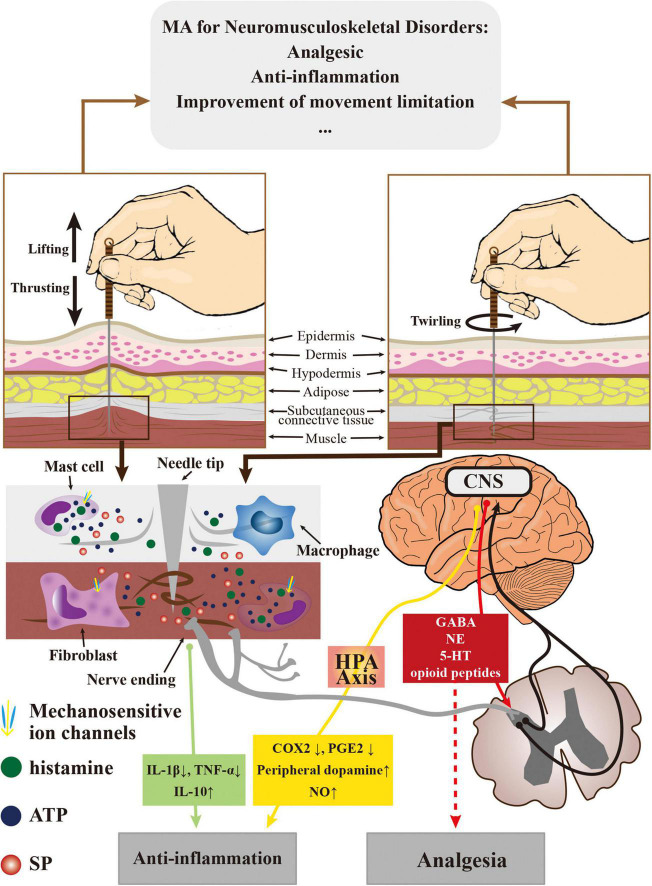
The effects of MA on neuromusculoskeletal disorders and the potential mechanism of action.

In addition to relieving the pain symptoms of neuromusculoskeletal disorders, MA can also downregulate the function of the hypothalamic-pituitary-adrenal (HPA) axis (Li et al., 2021), reduce the release of cyclooxygenase-2 (COX-2) and prostaglandin E2 (PGE2), promote the secretion of peripheral dopamine and nitric oxide (NO) (Wang M. et al., 2020), and relieve local inflammation (Yu et al., 2009). In the localized lesion (synovial fluid, cartilage, subchondral bone, etc.), MA can effectively inhibit the overexpression of inflammatory cytokines including interleukin-1β (IL-1β) and tumor necrosis factor α (TNF-α)and promote the expression of anti-inflammatory cytokine such as IL-10 (Wang M. et al., 2020). With the relief of local inflammation, the movement function of limbs can also be improved. Moreover, the result of a surface electromyographic (sEMG) study of shoulder joint dysfunction suggested that the improvement effect of MA on limb mobility may also lie in the enhancements of muscle excitability and endurance, as well as the delay of muscle fatigue (Wang I. et al., 2020; Figure 1).

## 3. Stimulation parameters of ma for selection

According to the operation methods of MA, the parameters closely related to the stimulation amount mainly include three types, namely, manipulation, kinematic and time parameters (Lyu et al., 2019). Different combinations of the above parameters will have corresponding stimulation amount and therapeutic effects on neuromusculoskeletal disorders (Yoon et al., 2022), which should be considered by acupuncturists.

### 3.1. Manipulation parameters

The selection of acupuncture manipulations mainly includes two basic needle movements with different operating directions. During treatment, the acupuncturist’s thumb and index finger operate the needle handle to move up and down or rotate on a fixed axis (the needle body), respectively (Seo et al., 2014; Xu et al., 2021). These two types of manipulations are named lifting-thrusting and twirling (Lyu et al., 2019). According to the movement mode of acupuncture needles, the mechanical stimulations of the acupoint area are also different. The stimulation of the lifting-thrusting method mainly pulls the epidermis, dermis, connective tissue and muscle through the frictional force along the needle body generated by the interaction between the needle and the surrounding tissue (Lee Y. S. et al., 2018; Yao W. et al., 2020), which significantly changes the morphological structure of the acupoint area, such as the thickening of connective tissues and the muscle layer (Bae et al., 2019). During this manipulation, the amplitude of needle movement can be regarded as the amplitude of the elastic body, and the resistance of the acupoint tissue to the needle can be regarded as the damping of the series of elastic bodies; therefore, such a process is similar to the Maxwell model (Wang, 2011). In terms of twirling, the needle body generates tangential frictional force with the surrounding tissue, which will induce the winding of connective tissues or muscle in the acupoint area and produce a stimulation effect (Yao W. et al., 2020). A clinical study found the sensation produced by lifting-thrusting is stronger than that of twirling (Lu et al., 2021). The result of an energy measurement experiment on animals also reported that the input average energy flux density of lifting-thrusting is greater than that of twirling. Therefore, if the operation time is the same, the stimulation amount of lifting-thrusting may be greater than that of twirling (Wang, 2011). Moreover, the stimulation amount of lifting-thrusting tends to increase with increasing needle diameter, which is a feature that twirling does not have (Lee Y. et al., 2018). However, due to the increased collagen winding or rupture caused by twirling, more cellular responses and release of active substances may be mediated by this process (Zhang et al., 2022).

At present, which manipulation should be selected in the treatment of different neuromusculoskeletal disorders and the applicable symptoms of each manipulation still lack evidence from rigorous RCTs. Therefore, the selection of manipulations in the clinical application of MA is still mainly based on the personal experience and judgment of acupuncturists. Although there are many clinical reports confirming that lifting-thrusting and twirling have positive effects in relieving pain and inflammation in musculoskeletal diseases (Comachio et al., 2020; Wang et al., 2021), according to some comparative studies of the two manipulations, twirling may be more suitable for local analgesia, regulation of blood circulation and anti-inflammation than lifting-thrusting, and the possible explanation for this result is related to its ability to release more neurotransmitters to regulate nerves (Xu et al., 2019). In terms of improving the movement function of joints, MA treatment is often accompanied by active movement of the patient to enhance the therapeutic effect, which is also called motion-style acupuncture. In the process of such treatment, MA often requires greater stimulation to obtain a strong needling sensation, thus, lifting-thrusting may be more suitable. For example, lifting-thrusting at the acupoint “Yanglingquan” (GB34) was selected in some clinical researches, and combined with flexion, extension and rotation of the patient’s joints with limited mobility to improve the joint movement functions of cervical spondylosis (Yang et al., 2022) and shoulder pain (Shi et al., 2018).

### 3.2. Kinematic parameters

The movement control of acupuncture needles by acupuncturists is mainly the adjustment of their kinematic parameters. In MA manipulation, the kinematic parameters of lifting-thrusting include the amplitude along the direction of the needle body and the corresponding velocity. Because these parameters of lifting-thrusting vary greatly among different acupoints, they need to be measured in future work. Take the acupoint “Quchi” (LI11) as an example, it was reported that the needling amplitude was usually around 0.9–1.8 cm, and the needling velocity was around 1.8–4.7 cm/s. The kinematic parameters of the twirling are the rotation angle with the needle body as the axis and the corresponding angular velocity. The common amplitude is between 180 and 360°, and the velocity is approximately 2.5–6 rad/s (Xu et al., 2021). At the same time, the ratio of the respective amplitude and velocity of these two types of manipulations is also the frequency of corresponding manipulations, so the needling frequency can also be adjusted by changing the needling amplitude or velocity (Lyu et al., 2019).

In general, the needling amplitude is most closely related to the stimulation intensity of acupuncture, and many acupuncturists adjust the intensity of stimulation by changing the needling amplitude. Some results of RCTs suggested that a greater needling amplitude may exerted better pain-relieving effects in neuromusculoskeletal disorders than a smaller amplitude (Zhang et al., 2021). For instance, the pressure pain threshold in a 10 cm needling amplitude increased higher than that in a 3 cm needling amplitude (Itoh et al., 2011). Similarly, the sensation of the *de qi* response as well as the pain threshold was also enhanced by a greater twirling angle (Choi et al., 2013). Although an excessively large needling amplitude may create potential risks, such as patient intolerance or neurological or vascular injury (Shen et al., 2021; Zhou et al., 2022), the result of an animal experiment suggested that the analgesic effect of MA may be positively correlated with the needling amplitude (Bae et al., 2019). The occurrence of this phenomenon probably lie in two factors. First, the excitation of afferent nerve fibers. Acupuncture activates baroreceptors, stretch receptors, and free nerve endings, which in turn transmit needling signals to the CNS from the afferent nerve fibers. Some animal studies have reported that small-amplitude MA mainly excites Aα and Aβ afferent fibers, while large-amplitude MA can excite all four types of afferent fibers (Aα, Aβ, Aδ, and C), thereby transmitting more signals and exciting more central nerves (Huo et al., 2020). Especially the twirling amplitude (angles) was reported to have an obvious dose-dependency on the cytoskeletal response in subcutaneous tissue when performing bidirectional rotation, which may also be due to the different winding and squeezing of afferent fibers at different twirling angles (Chang et al., 2019). Second, mechanosensitive ion channels are activated. As a typical mechanical stimulation, MA can activate non-selective mechanosensitive ion channels in the cell membrane during needling and regulate the conduction of needling signals, some key mechanotransducers, such as TRPV1 (Chen et al., 2018), TRPV2 (Huang et al., 2018), Piezo1, and Piezo2 (Guo et al., 2022), are involved in the regulation process. It was also found that the expression of related ion channel proteins exhibits varying degrees of stimulation intensity dependence (Chen et al., 2018; Chen C. et al., 2021). In contrast, this effect can be blocked by non-selective mechanosensitive channel blockers such as gadolinium (Varani et al., 2009). However, because the current real-time measurement technology of MA amplitude cannot meet the requirements of clinical application, and RCTs related to the therapeutic effect and mechanism of different needling amplitudes cannot be carried out. Therefore, it is necessary to focus on the development of MA quantification technology in future work.

Needling velocity is another important stimulation parameter for acupuncturists; moreover, the reinforcing and attenuating methods of acupuncture manipulation are also mainly distinguished according to the needling velocity (Lyu et al., 2019). Needling was able to arouse the deformation of the extracellular matrix and form a mechanical stress field in the interstitium, which hinged on needling velocity (Yao et al., 2018). This stress field causes winding or rupture of collagen fibers and muscles, and its magnitude affects the degree of cellular response around acupoints (Langevin et al., 2001). Although it was suggested that increasing the velocity of twirling (from 2 to 4 rad/s) was beneficial to relieve pain caused by neuromusculoskeletal disorders (Song et al., 2021), more clinical studies have pointed out that too fast or too slow needling velocity may cause a decline in therapeutic effect (Zhang L. et al., 2019). Therefore, the selection of an appropriate needling velocity is an important factor for obtaining a positive effect too. Acupuncture-related neurophysiological studies have proposed a possible explanation for this phenomenon. Since the increase in the needling velocity usually results in an increase in the MA frequency, with the change in synchronization of activated neural circuits, different frequencies of MA may form diverse neuronal connectivities (Yu et al., 2017), thus, the neuronal firing rate and time sequences of interspike intervals could be effectively distinguished at different MA frequencies (Zhou et al., 2014). The different acupuncture frequencies generated by different needle velocities can also regulate the above process, and the characteristics of rate encoding specific to different frequencies of MA have been found in related studies (Yu et al., 2017; Zhang L. et al., 2019). Nonetheless, because of the relative refractory period, the mean neuronal firing rates do not increase evidently when the frequency of MA is over 100 times per minute (Men et al., 2012). Therefore, although increasing the needling velocity can speed up neuronal firing and promote the rapid conduction of needling signals, a velocity that is too fast may not accelerate the above process, improve the therapeutic effect and also bring potential safety risks.

From the above results, it can be seen that the velocity of lifting-thrusting or twirling probably have a relatively effective reference range in the MA treatment of neuromusculoskeletal disorders, and the possible decrease in therapeutic effect or increased safety risk may be generated by too fast or too slow needling velocity outside this range. However, this reference range is likely to be vary according to the different disease locations or individuals. The acquisition of such evidence requires the accumulation of a large amount of clinical or experimental data, as well as the greater innovation and breakthroughs in measurement technology of MA kinematic parameters.

### 3.3. Time parameters

Complete MA treatment mainly includes three types of time parameters. The first one is needling time, which is the time when the acupuncturist uses his fingers to operate the acupuncture needles and perform manipulation. The second one is retention time. The process of needle retention is when needles stay underneath the skin after penetrating and are usually scheduled after needling is completed to consolidate the stimulation effect. The last one is course of treatment, a period of continuous MA therapy prescribed for specific diseases. According to current clinical studies, the common needling time ranges from 30 s to 5 min, the needle retention time usually ranges from 20 minutes to 1 hour, and the common course of treatment ranges from 2 weeks to 3 months (Lin et al., 2019; Lu et al., 2020).

In the process of MA treatment, the increase of needling time directly leads to an increase of the stimulation amount. An animal experiment comparing the effects of different needling times on neurophysiology have shown that the significant improvements of neurological function and cerebral blood flow were obtained by appropriately extending the needling time (from 5s to 60s). On the contrary, such effects were weakened when the needling time was extended to 180s (Zhang et al., 2015). Based on the above results, the remarkable thing is that there may be no necessary link between the increase in needling time and effect improvement. First, not all patients can tolerate an increased stimulation amount, especially thin female patients, so acupuncturists may need to adjust the other stimulation parameters to improve the therapeutic effect. Second, prolonged acupuncture stimulation probably lead to a weakening of effects, which is considered as a phenomenon called “acupuncture tolerance” (Han, 2011). The possible mechanisms of such a phenomenon may be related to not only the inactivation or downregulation of central opioid peptide receptors caused by excessive stimulation but also the release of anti-opioid peptide substances (such as cholecystokinin) (Han, 1995).

Although the needling stimulation was stopped during needle retention, needle retention can be regarded as an amplification effect on the needling stimulation. It has been found that the therapeutic effect of MA presents stage characteristics as retention time changes. An clinical fMRI study indicated that the related brain nuclei of the limbic system had obvious response patterns that changed over time during the effects of acupuncture, which could be divided into short activation, intermittent activation, bidirectional activation and continuous activation (Bai et al., 2009). Among them, continuous activation is mainly related to the post needling effect, and retention time is one of its main influencing factors. Numerous studies have confirmed that a better therapeutic effect can be obtained by retaining the needles for a certain period of time compared with direct needle withdrawal after needling, while an exceedingly long retention time may cause needle withdrawal resistance and tiresome acupoints (Lin et al., 2019). Moreover, for neuromusculoskeletal disorders, the evidence from an animal experiment showed the analgesic effect of 20 min of retention time for chronic pain is better than that of 10, 30, and 40 min (Cui J.-m Ma et al., 2009), while another clinical study suggested the MA treatment of acute pain can appropriately extend the needle retention time to 45 min (Yao and Liu, 2013).

As far as the course of treatment is concerned, due to the limited stimulation by a single MA treatment, it is necessary to continue such treatment for a period of time to accumulate effect. A study on rheumatoid arthritis showed that longer course of treatment could lead to better therapeutic effects (Wang et al., 2008). Similarly, it was found that better analgesia effect on fibromyalgia was produced by more MA treatments (Harris et al., 2005). However, acupuncture tolerance may also be resulted by an excessively long course of treatment, which has negative effects or even leads to more adverse events (Macpherson et al., 2004).

## 4. Discussion

Throughout the current research, MA has a relatively positive effect on the treatment of neuromusculoskeletal disorders. The selection of MA stimulation parameters can be summarized as follows according to the relevant evidence of its effects (Table 1). First, although both lifting-thrusting and twirling have analgesic and anti-inflammatory effects and improve movement limitation, lifting-thrusting may be more suitable for use in motion-style acupuncture to increase the movement amplitudes of joints, while twirling is probably more suitable for relieving pain and inflammation. Second, for the kinematic parameters, appropriately increasing the needling amplitude within a safe range is likely to bring about a certain enhancement in therapeutic effect, but the needling velocity should potentially have a certain effective reference range. Too large or small needling velocity is not conducive to obtaining better prognosis. Finally, the selection of time parameters is similar to that of needling velocity, and the time of needling and retention, as well as the course of treatment need to be adjusted according to different symptoms and individuals. In general, the selection of an excessively short time parameter probably tends to reduce the therapeutic effect because of an insufficient stimulation amount, whereas an excessively long time may not only do not contribute to the improvement of the therapeutic effect but also be prone to other side effects. In summary, it is not difficult to find that the selection of stimulation parameters in the clinical process of MA is still confusing and lacks evidence and a clear reference, which needs a large number of comparative clinical trials in future work.

**TABLE 1:** Common parameter types, values and corresponding effect of MA in the treatment of neuromusculoskeletal disorders.

Parameter type	Option	Common value	Potential parameter-effect relationship
Manipulation parameter	Lifting-thrusting	–	May be more suitable for improving joint movement
	Twirling	–	May be More suitable for analgesia and anti-inflammation
Kinematic parameter	Needling amplitude	Amplitude of lifting-thrusting: Varies by acupoints Amplitude of Twirling: 180–360°	A certain positive correlation between the needling amplitude and the effect may be existed
	Needling velocity	Velocity of lifting-thrusting: Varies by acupoints Velocity of Twirling: 2.5–6 rad/s	A certain effective reference range is probably existed Too slow: insufficient stimulation Too fast: no effect improvement and potential safety risks
Time parameter	Needling time	30 s–5 min	A certain effective reference range is probably existed Too short: insufficient stimulation Too long: patient intolerableness and acupuncture tolerance
	Retention time	20 min–1 h	A certain effective reference range is probably existed Too short: insufficient post needling stimulation Too long: needle withdrawal resistant and tiresome of acupoints
	Course of treatment	2 weeks–3 months	Accumulation of therapeutic effects of every MA treatment, and a certain effective reference range is probably existed Too short: insufficient accumulated stimulation Too long: patient intolerableness and acupuncture tolerance

**FIGURE 2:**
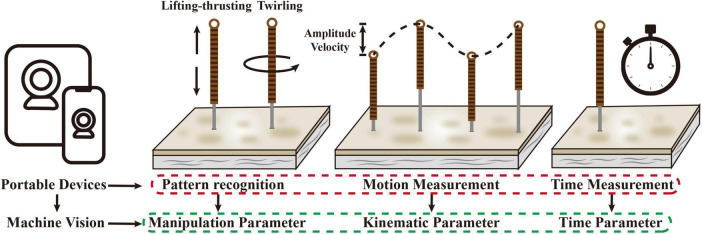
Recognition and measurement of stimulation parameters of MA based on machine vision.

Regarding the issue above, one of the main breakthroughs for the solution lies in the innovation of the measurement technology of kinematic parameters of MA. Currently, the measurement of MA stimulation parameters mainly includes two technologies. The first is the modification of the acupuncture needle body or needle handle. For example, a small kinematic sensor was attached to the needle handle to obtain the velocity and acceleration of the needle body during the needle movement (Seo et al., 2014; Leow et al., 2016), and the sensor device can also be wrapped around the needle body to obtain the needle movement amplitude and velocity (Davis et al., 2012; Li et al., 2013). Whether it is the modification of the needle handle or the needle body, the shape and weight of the acupuncture needle will be greatly changed. Therefore, this method has a serious influence on the finger sensation of acupuncturists and is not suitable for clinical application (Lyu et al., 2019). The other technology is motion tracking for capturing the movements of the thumb and index finger by placing small tracking markers (reflective balls) on the fingertips and joints of the acupuncturist’s fingers (Tang et al., 2018; Xu et al., 2021, 2022). Since the movement of the needle body is mainly generated by the lifting-thrusting or twirling of the thumb and index finger, the kinematic parameters of the needle can be calculated by tracking the movement of fingers. This technology completed real-time in-body parameter acquisition without operational interference and influenced the finger sensation. However, before the implementation of motion tracking technology, it is necessary to build an experimental environment for simultaneous shooting of multiple cameras. The configuration of multiple cameras, tripods and connecting wires still greatly affects the working environment of acupuncturists, which cannot actually be applied in clinical work. Therefore, the technology that is truly suitable for the stimulation parameter acquisition of MA should have the following characteristics: real-time, convenient, stable, and free of operational interference. According to the current development of motion analysis technology, the more suitable solution may be motion analysis technology based on machine vision. After the cameras captured the real-time video of the fingers and acupuncture needle during the operation of the acupuncturist, the image analysis technologies of machine vision such as convolutional neural networks could be used to identify the movement of the fingers or needles, determine the current manipulation mode (lifting-thrusting or twirling) (Su et al., 2022), and calculate the relevant kinematic parameters. Because this technology can be implemented with portable devices such as mobile phones and tablets, it may be a more suitable tool for the measurement of MA stimulation parameters for clinical applications (Figure 2).

Another breakthrough lies in the analysis of data. Because MA treatment requires the selection of the above three types of parameters to form a parameter combination for obtaining the therapeutic effect. Thus, compared with the single parameter analysis, different parameter combinations will significantly increase the complexity and difficulty of data analysis. Some studies have also focused on the influence of stimulation parameter interactions on the therapeutic effect. For instance, an investigation on the effects of parameter combinations, including manipulation (present or absent), retention time (1 or 21 min) and selected acupoints [“Hegu” (LI4) or non-acupoint located with the same dermatome of LI4], discovered that the combination of 21-min intervention on LI4 with manipulation showed better needling sensation and analgesic effect (Loyeung and Cobbin, 2013). Another study about the safety of manipulation by an auto manipulation device for acupuncture at different velocities and stimulation times suggested that although the stimulation time was shortened, more collagen rupture was brought about in higher velocity manipulation (Liu et al., 2018). These results indicated that different parameter combinations will result in too many intervention groups and corresponding effects, and traditional RCT experiments may be unable to meet the requirement of such research. On the basis of an innovative measurement solution suitable for clinical application, if this measurement method can be used in the clinical work and fundamental research of MA to obtain a large number of parameter combination data and the correspondence effects, some classification- and regression-related machine learning algorithms, such as decision tree (Ghiasi et al., 2020) and random forest (Wang S. et al., 2020), can be applied to find the different combinations of MA stimulation parameters with ideal therapeutic effects suitable for different neuromusculoskeletal disorders and individuals. We believe that with the continuous iteration of measurement and analysis technologies, the selection of MA stimulation parameters for neuromusculoskeletal disorders will be supported by more evidence and more standardized to achieve better therapeutic effects.

## 6. Conclusion

This article reviews three types of stimulation parameters that MA can select in the treatment of neuromusculoskeletal disorders and their corresponding effects. The manipulation parameters include lifting-inserting and twirling, both of which can achieve positive therapeutic effects. Lifting-thrusting may be more suitable for improving joint movement, while twirling is probably more preferred for analgesia and anti-inflammation. In terms of the kinematic parameters, appropriately increasing the needling amplitude within a safe range may bring about a certain enhancement in therapeutic effect, but the needling velocity is likely to have a certain effective reference range. A similar situation also exists in the selection of time parameters; too short- or too long-time settings may result in insufficient stimulation and acupuncture tolerance, respectively. At present, there is still a lack of sufficient experimental and clinical evidence to provide clear reference for MA application. It is urgent to pursue innovation in MA stimulation parameter measurement technology and data analysis algorithms, which may be the breakthroughs to solve this issue, and leading to a more standardized MA treatment for neuromusculoskeletal disorders.

## Author contributions

JX, GX, and W-CT developed and designed the study. B-GW and L-LX summarized and analyzed the current studies and wrote the manuscript. H-YY and W-CT reviewed and edited the manuscript. All authors read and approved the manuscript.
